# Supplementary material for: WordCommentsAnalyzer: A windows software tool for qualitative research
Source: F1000Res. 2018 Sep 4;7:536. Originally published 2018 May 3. [Version 2] doi: 10.12688/f1000research.14819.2 (PMC6137411; doi:10.12688/f1000research.14819.2)
Supplement: Supplementary file 3 [file f1000research-7-17614-s0002.tgz › b2a79496-4ada-47c4-9682-a99ce867836d.zip!/Eight Iranian Health Journals/Journal of Research in Rehabilitation Sciences _26.docx]

© Journal of Research in Rehabilitation Sciences . This work is licensed under CC BY-NC 4.0 (https://creativecommons.org/licenses/by-nc/4.0/).

Volume & Issue: ویژه نامه توانبخشی :1392

Title: تغییر پوسچر لگن در وضعیت ایستاده و تاثیرآن بر شاخص‌های اولتراسونیک کنترل ادرار در دوگروه زنان با و بدون بی اختیاری استرسی ادراری

http://jrrs.mui.ac.ir/index.php/jrrs/article/view/905

مقدمه:تاثیر تغییر وضعیت بدن بر عملکرد عضلات کف لگن در مطالعات محدودی گزارش شده است. به نظر می‌رسد این مطالعات به نوعی تاثیر مداخله به شکل تغییر یا اصلاح پوسچر را بر ثبات کف لگن و به کار گیری آن را در درمان بی‌اختیاری استرسی ادراری مطرح می‌کنند. هدف این مطا لعه بررسی تاثیر تغییر وضعیت ناحیه لگن، انقباض عضلات کف لگن و عرضی شکم بر سطح پایداری کف لگن و مقایسه آن در دو گروه زنان با و بدون بی اختیاری استرسی ادراری با استفاده از اولتراسونوگرافی به روش ترانس لبیال بود. مواد و روش‌ها: این مطالعه نیمه تجربی بر روی 41 زن (21زن مبتلا به بی اختیاری استرسی ادراری و20 زن بدون هیچ نوع بی اختیاری ادراری ) مراجعه کننده به درمانگاه زنان بیمارستان ولی عصر-م جتمع بیمارستانی امام خمینی تهران انجام گرفت. پس از تکمیل فرم اطلاعاتی، تصویربرداری به روش ترانس لبیال در وضعیت‌های ایستاده با حفظ وضعیت نوترال لگن، تیلت قدامی وخلفی لگن و حین انقباض عضلات کف لگن و عرضی شکم انجام شد. سپس شاخص‌های اولتراسونیک معرف سطح پایداری کف لگن مانند زوایای آلفا و بتا و فاصله H اندازه گیری شدند.از نرم افزارآماری SPSS نسخه 16 و آزمون‌های کولمگروف –اسمیرنف، تی مستقل‌، آنالیز واریانس با تکرار یک طرفه، آزمون تعقیبی مقایسه زوج‌های بونفرونی و پیرسون برای تحلیل داده‌ها استفاده و میزان P کمتر از 05/0 معنی‌دار در نظر گرفته شد. یافته‌ها: بررسی تاثیر تعامل مداخله و گروه بر شاخص‌های اولتراسونیک نشان داد که اثر اصلی تغییر وضعیت بر روی زوایای آلفا، گاما و صفحه لواتورا معنی‌دار بود(05/0>P) ولی تاثیری برزاویه بتا نداشت (05/0<P). مقایسه بین دوگروه نشان داد که حین تیلت خلفی لگن میانگین زاویه گاما و زاویه صفحه لواتور بین دوگروه تفاوت معنی‌دار داشت با (05/0>P). در مقایسه تیلت قدامی و تیلت خلفی، تیلت قدامی در هر دو گروه سبب افزایش زاویه گاماشده بود (05/0>P). نتیجه‌ گیری: بر اساس نتایج این مطالعه تیلت خلفی در مقایسه با تیلت قدامی به عنوان وضعیتی که سبب تغییر شاخص‌های اولتراسونوگرافیک در جهت افزایش پایداری مجموعه کف لگن می‌شود، شناخته می‌شود.

Title: سیری از خانه تا جامعه با چالشهای هندلینگ در کودکان مبتلا به فلج مغزی: تحلیل محتوی کیفی

http://jrrs.mui.ac.ir/index.php/jrrs/article/view/1134

مقدمه: مشکلات کودکان فلج مغزی طولانی مدت و دائمی است و شناخت چالش‌های آنان، مستلزم آگاهی از ساختار محیطی و بافتار فرهنگی است. لذا هدف از این مطالعه، تبیین چالش‌های هندلینگ کودکان فلج مغزی از دیدگاه کودکان فلج مغزی، مادرانشان و کاردرمانگران در جامعه ایرانی می باشد. مواد و روش‌ها: این مطالعه کیفی، بخشی از مطالعه گسترده در مورد چگونگی هندلینگ در کودکان فلج مغزی ایرانی است که با مشارکت دو کودک فلج مغزی، چهار مادر کودک فلج مغزی و چهار کار درمانگر انجام گردید. افراد طبق نمونه گیری هدفمند و در ادامه نمونه‌گیری نظری انتخاب شدند. داده‌ها با استفاده از مصاحبه عمیق فردی و گروهی به صورت نیمه ساختارمند و مشاهده هدفمند در منزل و کلینیک جمع آوری شدند و به روش تحلیل محتوی عرفی مورد تجزیه و تحلیل قرار گرفتند. یافته‌ها: از مجموع مصاحبه‌ها، 1275 کد به دست آمد. این کدها در طی فرایند تجزیه و تحلیل در چهار درونمایه اصلی، هندلینگ در منزل، هندلینگ در کلینیک، هندلینگ در مدرسه و هندلینگ در جامعه به عنوان محیط‌های اصلی چالش کودکان فلج مغزی و نیز 11 زیر درونمایه طبقه بندی شدند. نتیجه‌گیری: مشارکت کنندگان، ضعف آموزش‌های عملی را اصلی‌ترین چالش هندلینگ در منزل، توجه صرف به تمرینات درمانی و عدم مشارکت فعال کودک در تمرینات درمانی را مشکل اصلی در کلینیک و مراقبت محور نبودن هندلینگ و سوء استفاده را به عنوان چالش‌های اصلی هندلینگ در مدرسه و جامعه عنوان کردند که تحقیقات بیشتری در مورد مناسب سازی، نحوه مراقبت از کودکان فلج مغزی و آموزشهای هندلینگ در خانه، کلینیک، مدرسه و جامعه مورد نیاز است.

Title: تاثیر برنامه درمان نوای گفتار بر وضوح گفتار بیماران مبتلا به پارکینسون

http://jrrs.mui.ac.ir/index.php/jrrs/article/view/1336

مقدمه: پارکینسون یک بیماری نرولوژیک پیشرونده است، که برروی حرکات غیر گفتاری و جنبه های مختلفی از تولید گفتار اثر گذاشته و باعث بروز نقص در تنفس، آوا سازی، تولید و نوای گفتار شده و  در مجموع منجر به کاهش وضوح گفتار و در نتیجه مختل شدن ارتباط می گردد. هدف اصلی این پژوهش بررسی اثر برنامه درمان نوای گفتار بر وضوح گفتار سه بیمار مبتلا به پارکینسون است. مواد و روش ها: در مرحله اول تکالیف برنامه درمانی متناسب با زبان فارسی و با حفظ اولویت درمانی برای بیماران مبتلا به پارکینسون طراحی و در اختیار ده آسیب شناس گفتار و زبان خبره جهت تعیین روایی محتوایی  قرار داده شد. پس  از تدوین نهایی برنامه اثر آن بر وضوح گفتار سه بیمار مبتلا به پارکینسون مورد بررسی قرار گرفت. یافته ها: برنامه درمانی طراحی شده  با CVR ≥0/62   و CVI =0/94  مورد تایید آسیب شناسان گفتار و زبان قرار گرفت. سپس نتایج حاصل از اجرای آن با استفاده از ترسیم نمودار، تحلیل چشمی ، محاسبه اندازه اثر دی کوهن و درصد بهبودی مورد بررسی قرار گرفت. اندازه اثر در هر سه بیمار بیشتر از 8/0 بود و همه بیماران درصدی بهبودی بالینی به دنبال دریافت برنامه درمان  نیز نشان دادند. نتیجه گیری: طبق نتایج بدست آمده، برنامه درمانی طراحی شده منجر به افزایش وضوح گفتار هر سه بیمار شده و نتایج درمانی آن به مدت سه  الی چهار هفته نسبتا پایدار بوده است.

Title: بررسی تأثیر انواع پانسمان و مدیریت بعد از عمل بر آماده‌سازی استمپ در قطع عضو زیر زانو

http://jrrs.mui.ac.ir/index.php/jrrs/article/view/998

مقدمه: از دست دادن اندام تحتانی به هر علتی منجر به ایجاد مشکلات فردی و اجتماعی فراوان برای فرد دچار قطع عضو می‌گردد. مراقبت صحیح از استمپ بعد از عمل جراحی با کمک انواع پانسمان نقش مؤثری در کسب نتایج مطلوب داشته و سبب کاهش مشکلات استمپ و آمادگی هر چه سریع‌تر برای دریافت پروتز می‌شود. اما در بررسی‌های انجام گرفته، نتایج متفاوتی در مورد بهترین نوع پانسمان ارائه شده است. بنابراین در مطالعه حاضر با مرور بر مقالات موجود، سعی بر آن است که بهترین نوع پانسمان معرفی شود. مواد و روش‌ها: در مطالعه حاضر برای رسیدن به هدف، با مراجعه به متون منتشر شده در پایگاه‌های اطلاعاتیScience Direct، Google cholar و Pubmed، با استفاده از کلمات کلیدی Prosthesis, Post Operative Care, Rigid & Soft dressing, Below knee amputation، اطلاعات مربوط به انواع پانسمان و مدیریت بعد از عمل بر آماده‌سازی استمپ در قطع عضو زیر زانو جمع‌آوری شد و نتایج آن‌ها مورد بررسی قرار گرفت. جهت ارزیابی مقالات از ابزارDowns and Black  استفاده گردید. یافته‌ها: پس از اتمام جستجو بر اساس کلمات کلیدی، تعداد 123 مقاله به دست آمد که در نهایت تعداد 15 مقاله با توجه به معیارهای انتخاب برای تحلیل نهایی پذیرفته شدند. نتیجه‌گیری: در پایان مقاله حاضرانواع مطالعات ازRCT  تا Case Report را بررسی نموده است. نتایج نشان داده است که پانسمان‌ سخت در مقایسه با پانسمان‌ نرم در آماده‌سازی استمپ زیر زانو و بهبود مشکلات آن بهتر عمل می‌کند. اکثر مطالعات، برمراقبت از استمپ بعد از عمل جراحی، کسب نتایج مطلوب و کاهش عوارض آن در کوتاه‌ترین زمان ممکن تأکید داشتند. البته در مورد بازیابی عملکرد و توانایی راه رفتن توسط پروتز اطلاعات بسیار محدود است و پیشنهاد می‌گردد به علت اهمیت این موضوع مطالعات بعدی بر این موارد متمرکز گردند.

Title: تاثیر تمرینات کف لگن بر بهبود بی‌اختیاری ادرار در زنان: مقاله مروری

http://jrrs.mui.ac.ir/index.php/jrrs/article/view/1232

بی اختیاری ادرار(urinary incontinence یا UI) یک مشکل شایع در بین افراد بزرگسال جامعه است و اغلب درخانمها مشاهده می شود. با افزایش سن, تعداد حاملگی و افزایش BMI، شانس ابتلا بیشتر می شود. درمان های کانزرواتیو معمولا به عنوان اولین خط درمان پیشنهاد میشوند. مداخلات فیزیوتراپی که در راس آن ها تمرینات پلویک فلور هستند جز اصلی همین درمان های کانزرواتیو به حساب می آیند .هدف ما از این مطالعه جمع اوری وذکر تمرینات وتکنیک های موثر در درمان و بهبود بی اختیاری ادرار است . بیان این نکات به درمانگران فیزیکی کمک خواهد کرد تا به درمان موثرتر این عارضه بپردازند. مواد و روش ها: پژوهشگر حاضر با مراجعه به متون منتشر شده در دودهه اخیر در پایگاه های اطلاعاتی PEDro،Science direct ، Pubmed نسبت به جمع آوری داده ها واعلام نتایج ان ها اقدام کرده است. یافته ها: نتایج بدست آمده از مقالات منتشر شده در طی در دو دهه ی اخیر نشان می دهند که استفاده از تمرینات پلویک فلور در بهبود بی اختیاری ادرار در زنان بسیار موثر هستند. نتیجه گیری: در اکثرمطالعات صورت گرفته، بهبودی در طول مدت درمان وبلافاصله پس از قطع آن بررسی شده است. این در حالی است که نیاز به تحقیقات بیشتری پیرامون تاثیرات دراز مدت درمان وجود دارد  تا روند بهبودی را پس از پایان دوره ی تمرینات کف لگن بصورت گسترده تری بررسی نماید.

Title: تجارب افراد با فلج مغزی از توانبخشی: بررسی مروری

http://jrrs.mui.ac.ir/index.php/jrrs/article/view/1251

مقدمه: فلج مغزی یک ناهنجاری غیرپیشرونده در مغز در حال رشد است، كه نيازمند خدمات وسيع توانبخشي به صورت طولاني مدت مي‌باشند. تجربه از درمان، قسمت عمده‌ای از تجارب این افراد را تشکیل می‌دهد. درک تجارب افراد با فلج مغزی از خدمات توانبخشی، در بهبود نگرش ارایه دهندگان این خدمات از اهمیت خاصی برخوردار است. مواد و روش‌ها: این مطالعه یک پژوهش مروری است. برای یافتن مطالعات کمی و کیفی مرتبط با هدف پژوهش، جست‌وجو در پایگاه‌های انگلیسی GoogleScholar، PubMed، Medline، Eric، Psychinfo، OVID، Cochrane library، CINAHL، Web of Knowledge، PEDRO، OTseeker، Rehabdata، Rehadatو پایگاه های فارسی SID، Iranmedex، Noormags، Medlib،Magiran وIrandocدر بازه زمانی سال‌های 1980 تا 2012 انجام گرفت و در نهایت 15 مطالعه (13 مطالعه کیفی و 2 مطالعه کمی نظرسنجی) مرتبط یافت شد. یافته‌ها: با بررسی مطالعات یافت شده، دو درونمایه اصلی شامل «تجارب درک شده از درمان» با زیردرونمایه‌های احساسات جسمانی و روانشناختی از درمان؛ محیط درمان و وسایل مورد استفاده؛ لذت، انگیزه، معناداری و هدف در درمان؛ امنیت؛ درمان مراجع محور؛ درمان‌های رایج و غیررایج؛ نقش اطرافیان فرد در تجربه از درمان و سیستم درمانی؛ و همچنین «تجارب ناشی از اثرات درمان» با زیردرونمایه‌های برآیند جسمانی درمان؛ برآیند روانشناختی درمان و اثربخشی درمان بر جنبه‌های مختلف زندگی به دست آمد. نتیجه‌گیری: درمانگران و محققین نیازمند توجه به رضایتمندی در تجارب درک شده از درمان و تجارب ناشی از اثرات درمان در افراد با فلج مغزی هستند. تحقیقات بیشتری به منظور تبیین تجارب افراد با فلج مغزی از خدمات ارائه شده ضروری به نظر می‌رسد.

Title: دستگاه وابران شنوایی و مهار دگرسوی گسیل‌های صوتی گوش

http://jrrs.mui.ac.ir/index.php/jrrs/article/view/1329

مقدمه: از زمان کشف گسیل‌های صوتی گوش یا OAEs (otoacoustic emissions)، پیشرفت‌های شگرفی در زمینه‌ ثبت این پاسخ در گروه‌های بالینی مختلف و در بررسی فیزیولوژی حلزون صورت گرفته است. یکی از مهمترین کاربردهای OAEs، بررسی عملکرد دستگاه وابران شنوایی است. این مقاله به فیزیولوژی دستگاه وابران شنوایی و پیامدهای درگیری این دستگاه در جمعیت‌های مختلف بالینی می‌پردازد. بر خلاف دستگاه آوران شنوایی که اطلاعات فراوانی از آن در دسترس است، در رابطه با دستگاه وابران شنوایی، اطلاعات اندکی در دسترس است و همین مسئله کاربرد بالینی این آزمون شگرف را کاهش داده است. مواد و روش‌ها: این مقاله با مرور بر روی 40 مقاله‌ی جامع در خصوص آزمون OAEs و مهار وابران شنوایی تهیه شده است. جستجوی مقالات در pubmed، sciencedirect ، google scholar و proquest صورت گرفت و مقالات جامع با روش بررسی مناسب، که شامل نتایج بررسی دستگاه وابران در جمعیت‌های بالینی بود، برای این مقاله‌ی مروری انتخاب شدند. یافته‌ها: در مورد عملکرد و ویژگی‌های LOCB(Lateral Olivocochlear Bundle) اطلاعات اندکی در دسترس است. اخیرا پیشنهاد شده است که رشته‌های LOCB در ایجاد و حفظ عملکرد هنجار حلزون نقش مهمی ایفا می‌کند. دستگاه وابران به عنوان یک دستگاه تنظیم‌کننده، امکان کنترل مراکز بالاتر مغزی را روی فرآیندهای فعال حلزونی (همانند الکتروموتیلیتی OHCs) فراهم می کند. همچنین فعال شدن COCB اثر ضد پوشش دارد و نسبت سیگنال به نویز را در دستگاه شنوایی افزایش می‌دهد.بنابراین باعث بهبود شنوایی در حضور نویز زمینه می‌شود. نتیجه‌گیری: OAE شاخص غیرتهاجمی، ساده و کارای بررسی تمامیت عملکرد حلزون است و آزمون مهار دگرسویی OAE ابزار بالینی غیرتهاجمی و عینی را جهت ارزیابی دستگاه وابران شنوایی در انسان‌ها فراهم می‌کند . کاربرد این آزمون در بیماران دچار اختلالات شنوایی رو به افزایش است. متخصصان با کسب اطلاع از نتیجه‌ی این آزمون در اختلالات شنوایی می‌توانند به تشخیص به موقع و توانبخشی بیماران کمک کنند.

Title: تحلیل تغییرات طول و نیروی تولیدی برخی از عضلات تنه در یک بیمار مبتلا به اسکولیوز ایدیوپاتیک هنگام راه رفتن با ارتز میلواکی با استفاده از نرم افزار -Open-simm پژوهش موردی

http://jrrs.mui.ac.ir/index.php/jrrs/article/view/962

مقدمه: اسکولیوز یک ناهنجاری سه بعدی ستون فقرات است که با انحراف جانبی وچرخش مهره‌ها همراه است. روش‌های مختلفی برای ارزیابی فعالیت عضلات تنه بیماران طی استفاده از ارتز هنگام راه رفتن به کار رفته است هر چند چگونگی تأثیرارتز برطول ونیروی تولید شده توسط عضلات تنه تاکنون مورد بررسی قرارنگرفته است. هدف از مطالعه حاضر بررسی تأثیر ارتز میلواکی بر طول و نیروی تولید شده توسط برخی عضلات تنه در یک بیمار مبتلا به اسکولیوز هنگام راه رفتن می‌باشد. مواد و روش‌ها: یک دختر 12 ساله دچار اسکولیوز در این مطالعه شرکت نمود. برخی از پارامترهای ارزیابی شده در این مطالعه شامل طول عضلات تنه، نیروهای تولید شده توسط آن‌ها و نیروی عکس‌العمل مفاصل هیپ وناحیه کمرمی‌باشند. نرم افزارهای Open SIMMو Visual 3D‌برای مدل کردن سیستم اسکلتی عضلانی بیمار به کار رفتند. یافته‌ها: طول عضلات راست کننده ستون فقرات(ارکتور اسپاین) با استفاده از ارتز افزایش یافت(27/13 درصد). هم چنین، نیروی تولید شده توسط عضلات تنه هنگام راه رفتن در دو وضعیت استفاده و عدم استفاده از ارتز و نیز بین دو سمت راست و چپ متفاوت بود(جدول5) . نتیجه‌گیری: در مطالعات قبلی از این نرم افزار به منظور اندازه‌گیری طول عضلات در اندام فوقانی یا تحتانی استفاده گردیده است و به طور ویژه ‌طول عضلات و نیروی اعمالی آن‌ها در بیماران دچار اسکولیوز با استفاده از این نرم افزار تاکنون مورد ارزیابی قرار نگرفته است. بر اساس نتایج حاصل از این مطالعه،به نظر می‌رسد نرم افزارOpen-SIMM قابلیت کاربرد در اندازه‌گیری طول و نیروهای فعال و غیرفعال اعمال شده توسط عضلات تنه در بیماران دچاراسکولیوز را هم دارد بنابراین پیشنهاد میگردد که این مطالعه برروی  نمونه آماری بزرگترانجام بگیرد.
